# Particulate Air Pollution Exposure and Stroke among Adults in Israel

**DOI:** 10.3390/ijerph20021482

**Published:** 2023-01-13

**Authors:** Britney Gaines, Itai Kloog, Inbar Zucker, Gal Ifergane, Victor Novack, Carmit Libruder, Yael Hershkovitz, Perry E. Sheffield, Maayan Yitshak-Sade

**Affiliations:** 1 Department of Environmental Medicine and Public Health, Icahn School of Medicine at Mount Sinai, New York, NY 10029, USA; 2 Department of Geography and Environmental Development, Ben Gurion University, Beer Sheva 8410501, Israel; 3 Ministry of Health, Jerusalem 9446724, Israel; 4 Negev Environmental Health Research Institute, Soroka University Medical Center, Beer Sheva 8410101, Israel; 5 Neurology Department, Soroka University Medical Center, Beer Sheva 8410101, Israel; 6 Clinical Research Center, Soroka University Medical Center, Beer Sheva 8410101, Israel; 7 Faculty of Health Sciences, Ben-Gurion University of the Negev, Beer Sheva 8410501, Israel; 8 Department of Pediatrics, Icahn School of Medicine at Mount Sinai, New York, NY 10029, USA

**Keywords:** air pollution, stroke, PM_2.5_

## Abstract

Stroke is the second most common cause of death and disability in the world. Many studies have found fine particulate matter (PM_2.5_) exposure to be associated with an increased risk of atherosclerotic cardiovascular disease, mostly focusing on ischemic heart disease and acute myocardial infarction. In a national analysis conducted in Israel—an area with unique climate conditions and high air pollution levels, we estimated the association between short-term PM_2.5_ exposure and ischemic stroke, intracerebral hemorrhage (ICH), or transient ischemic attacks (TIA). Using the Israeli National Stroke Registry, we obtained information on all stroke cases across Israel in 2014–2018. We obtained daily PM_2.5_ exposures from spatiotemporally resolved exposure models. We restricted the analytical data to days in which PM_2.5_ levels did not exceed the Israeli 24 h standard (37.5 µg/m^3^). We repeated the analysis with a stratification by sociodemographic characteristics and comorbidities. For all outcomes, the exposure–response curves were nonlinear. PM_2.5_ exposure was associated with a higher ischemic stroke risk, with larger effect estimates at higher exposure levels. Although nonsignificant, the exposure–response curve for TIA was similar. The associations with ICH were nonsignificant throughout the PM_2.5_ exposure distribution. The associations with ischemic stroke/TIA were larger among women, non-Jewish individuals, older adults, and individuals with diabetes, hypertension, and ischemic heart disease. In conclusion, short-term PM_2.5_ exposure is associated with a higher risk for ischemic stroke and possibly TIA, even when PM_2.5_ concentrations do not exceed the Israeli air quality guideline threshold. Vulnerability to the air pollution effects differed by age, sex, ethnicity, and comorbidities.

## 1. Introduction

Stroke is the second most common cause of death and disability in the world [1]. Global trends show an increase in stroke prevalence by 85%, incidence by 70%, and mortality by 43% [1]. Research in recent decades has shown the contribution of environmental exposures to stroke risk, beyond clinical and behavioral risk factors [2]. Across several studies there is evidence that air pollution exposure is associated with an increase in 2% to 21% excess risk of stroke [2]. Both long-term and short-term exposure to air pollution has been linked to stroke risk with the most pronounced associations found with ischemic stroke [3]. PM_2.5_ can be inhaled into the lower faculties of the lungs causing damage to the surrounding cells and leading to an inflammatory immune response [4]. Inhaling PM_2.5_ can cause damage to the central nervous system at large, resulting in oxidative stress and neuroinflammation which are precursors to neuropathologies such as stroke [5]. Finally, PM_2.5_ exposure was found to cause vascular damage and increase cardiovascular risk [6,7].

Ambient air pollution contributes to approximately 4.2 million deaths annually, and has been linked to atherosclerotic cardiovascular outcomes, and specifically stroke, in many studies [2,3,8,9]. For example, a 2019 meta-analysis found that each 5 µg/m^3^ increase in chronic fine particulate matter (PM_2.5_) exposure was associated with an 11% increase in ischemic stroke risk [10]. However, current research has several gaps. First, most studies do not distinguish between intracerebral hemorrhage (ICH) and ischemic stroke. Second, studies often lack information on important confounders and modifiers. Additionally, many studies used monitoring sites or coarse-gridded models to estimate air pollution exposure. This does not take spatial variation into account and can lead to exposure measurement error and biased effect estimates. Finally, cohorts used to identify the air pollution association with stroke are often limited in sample size or lack generalizability [11]. Thus, further research is required to support the causal association between PM_2.5_ exposure and stroke.

In many studies, certain populations were found to be more susceptible to air-pollution-related health effects. Studies in Israel found larger air pollution effects on respiratory outcomes among the non-Jewish population [12,13], possibly attributed to higher exposure levels due to housing conditions, or a higher prevalence of lower socio-economic status. Additionally, many studies found older adults and people with chronic conditions to be more susceptible to air-pollution-related cardiovascular outcomes [14,15,16,17]. A vulnerability assessment in air pollution studies is of great significance as it can guide public health departments in tailoring interventions to specific communities.

We analyzed the association between PM_2.5_ exposure and stroke in a national population-based study in Israel. Israel is in Western Asia and is surrounded by the Sahara Desert [18], Middle Eastern deserts, and the Mediterranean Sea. Average annual PM_2.5_ levels ranged from 21 to 25 µg/m^3^ in the last two decades. These levels are considerably higher compared to other developed countries. For example, the mean annual PM_2.5_ levels in the United States ranged from 7 to 10 µg/m^3^ in the last two decades [19]. The PM_2.5_ composition differs across Israel. In dense cities such as Tel Aviv, traffic is a major air pollution source. In Southern Israel, natural dust is a major source. Haifa has a higher concentration of pollutants originating from industrial sources, as well as from the combustion of fuel oil and the combustion of residual oils used by ships [18,20]. We utilized the national Israeli stroke registry (INSR), which includes detailed health information on all stroke cases across Israel, and highly spatiotemporally resolved exposure models to minimize measurement error. With these data, we assessed the association between short-term PM_2.5_ exposure and ischemic stroke, ICH, or transient ischemic attacks (TIA), and identified populations more vulnerable to the PM_2.5_ effect.

## 2. Materials and Methods

### 2.1. Study Population and Outcome

In this retrospective cohort study, we utilized the INSR to identify all stroke cases of individuals 18 years and older across Israel between the years 2014 and 2018. The INSR was formed in 2014 and includes data on all permanent residents in Israel admitted to a general hospital with a final International Classification of Disease, ninth edition (ICD-9) diagnosis of ischemic stroke (ICD-9 433, 434, 436, or 997.02), ICH (ICD-9: 431), or TIA (ICD-9: 435). Data are retrieved from electronic medical records of inpatient hospital visits quarterly and contains sociodemographic information (age, sex, ethnicity, and geocoded home or street address) and clinical information (subtype of stroke, anticoagulants, lipid-modifying treatment, comorbidities, obesity, and postevent treatments). Information on sociodemographic variables was retrieved from the Ministry of Interior and linked to the INSR. Ethnicity was grouped into Jewish versus non-Jewish due to the small percentage of the non-Jewish population. The non-Jewish group includes Arab, Druze, or other ethnic groups. Sex was coded as male or female. Age was calculated in years using the year of birth. A universal coded ID was available for each participant and allowed us to link demographic and clinical data and track repeated events. We excluded 2036 repeated events within the study period (2.2%) to avoid bias due to a differential baseline risk. We additionally excluded 1289 individuals without a valid residential address (1.6%).

### 2.2. Exposure

We obtained PM_2.5_ and temperature estimates from satellite-based spatiotemporal models that apply a hybrid approach and estimate daily mean temperature and PM_2.5_ exposures at a fine resolution [21,22]. Briefly, these models apply a hybrid modeling approach that consists of three stages, enabling spatially continuous PM_2.5_ or temperature estimations at a 1 × 1 km spatial resolution. The models were calibrated daily by applying a mixed-modeling approach using the MODIS-based multiangle implementation of atmospheric correction (MAIAC) aerosol optic depth (AOD) and various spatial and temporal predictors. This resulted in excellent performance with out-of-sample cross-validated R^2^ values of 0.82–0.92 for the PM_2.5_ model and 0.966–0.986 for the temperature model. Exposures were linked to each individual based on proximity to geocoded residential address.

To portray the potential risks posed by air pollution exposure that occurred within the range of acceptable air quality levels, our study was restricted to days in which the average PM_2.5_ levels did not exceed the Israeli air quality standard of 37.5 µg/m^3^. We linked the PM_2.5_ and temperature exposures based on a participant’s residential address. Based on previous studies, we defined the window of exposure as exposure on the same day and additionally assessed the associations with exposures 1–2 days before the event.

### 2.3. Statistical Analysis

We assessed the association between short-term PM_2.5_ exposure and stroke using a conditional logistic regression in a case crossover approach [23]. This approach controls for confounders that do not vary within a short period by design. We assessed the associations with ischemic stroke, ICH, and TIA separately. We defined the stroke event days as case days. The control days were selected within the same month and year and were matched by the weekday. We then assessed the associations between PM_2.5_ exposures at lag days 0, 1, or 2 with each of the outcomes, with adjustment for temperature exposure at the same exposure window as PM_2.5_. To allow for nonlinear associations, we repeated the models using a spline function for the PM_2.5_ exposure. As a sensitivity analysis, we further restricted the data to individuals with exact address information (rather than residential street information), to make sure our results were not biased due to exposure measurement error.

As a secondary analysis, we created subcohorts of sociodemographic groups (men versus women, Jewish versus non-Jewish, neighborhood socioeconomic score below or above the median, age below and above the median) and comorbidities (individuals with and without diabetes, hypertension, hyperlipidemia, and ischemic heart disease) to identify populations that were more vulnerable to the PM_2.5_ associations with ischemic stroke/TIA. Since we did not observe an association with ICH, and since the exposure–response curves of the associations with ischemic stroke and TIA were similar, we pooled these cases together for the purpose of this secondary analysis.

## 3. Results

We included 74,052 cases, of which 64.8% were ischemic stroke, 7.7% ICH, and 27.6% TIA. About 46% were women, the mean age was 71, and 80.9% were Jewish. Diabetes, hypertension, and hyperlipidemia were highly prevalent (Table 1). PM_2.5_ concentrations did not exceed the 24 h Israeli standard (37.5 µg/m^3^) in 97% of the days during 2014–2018. During these days, the average daily mean PM_2.5_ concentration was 17.8 µg/m^3^, with an interquartile range (IQR) of 6.3 µg/m^3^. The average daily mean temperature was 21.3 °C, with an IQR of 9.8 degrees.

Using linear terms, an IQR increase (6.3 µg/m^3^) in PM_2.5_ exposure on the event day was associated with a 1.7% increase in the risk for ischemic stroke (95% CI 0.15%; 3.28%), a 2.7% increase in the risk for TIA (95% CI 0.37%; 5.17%), and a 4.4% decrease in the risk of ICH (95% CI −8.57%; −0.09%). We did not find associations between PM_2.5_ exposure in lag days 1–2 and either of the outcomes (Table 2).

In a sensitivity analysis, we restricted the data to the 71.1% individuals who had exact address information and excluded the 29.9% with residential street information. The effect estimates in the restricted data were very similar to those observed in the full analytical dataset (Table 3).

**Figure 1:**
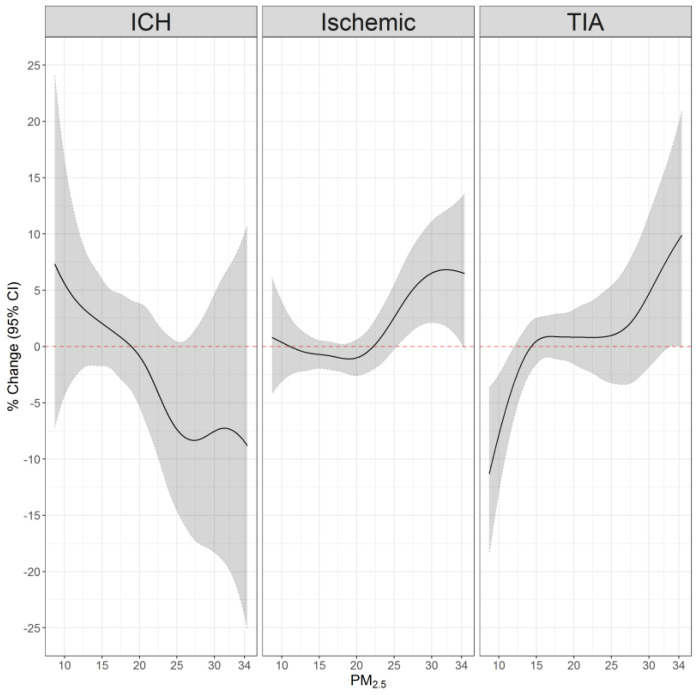
The association between PM_2.5_ exposure on the event day and types of strokes. Figure 1 shows the associations between PM_2.5_ exposure on the event day and intracerebral hemorrhage (ICH), ischemic stroke, or transient ischemic attack (TIA). Results were obtained from conditional logistic regressions, in which control days were selected within the same month and year as the case day and matched by the weekday. Models were adjusted for a spline function of daily mean temperature.

To explore potential nonlinear associations, we repeated the models using penalized spline functions to assess the association between exposure at the day of the event and each type of stroke. For all outcomes, the associations were nonlinear. PM_2.5_ exposure was significantly associated with a higher ischemic stroke risk, with larger effect estimates at higher exposure levels. Although nonsignificant, the exposure–response curve for TIA was similar. The associations with ICH were nonsignificant throughout the entire PM_2.5_ exposure distribution (Figure 1).

**Figure 2:**
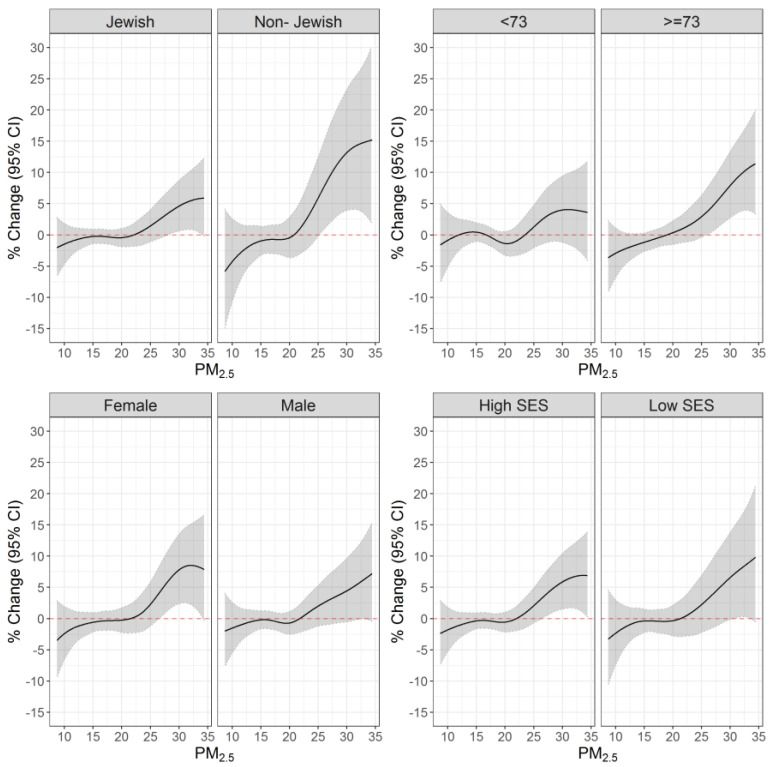
The association between PM_2.5_ exposure on the event day and ischemic stroke/TIA stratified by age, sex, ethnicity, and SES. Figure 2 shows the associations between PM_2.5_ exposure on the event day and ischemic stroke/transient ischemic attack (TIA), stratified by ethnicity (Jewish versus non-Jewish), median age (<73 years versus 73 years and older), sex (male versus female), and neighborhood socioeconomic status (SES) (lower than the median score versus median score or higher). Results were obtained from conditional logistic regressions, in which control days were selected within the same month and year as the case day and matched by the weekday. Models were adjusted for a spline function of daily mean temperature.

After pooling the PM_2.5_ exposure–response curves for ischemic stroke and TIA we assessed whether the PM_2.5_ exposure–response curves differ by sociodemographic characteristics and comorbidities. In all subcohorts, we observed larger effect estimates in higher exposure concentrations. The PM_2.5_ effects were especially pronounced among women, non-Jewish individuals, and older adults (Figure 2).

For example, the percent increases in ischemic stroke/TIA risks (95% CI) associated with a 1 µg/m^3^ increase in PM_2.5_ exposure when the reference was set up at 30 µg/m^3^, were: 4.61% (0.63%; 8.74%) and 13.18% (3.90%; 23.30%), among Jewish vs. non-Jewish, 3.97% (−1.19%; 9.42%) and 7.92% (2.75%; 13.36%) among younger vs. older adults, and 4.44% (−0.52%; 9.65%) and 7.80% (2.40%; 13.50%) among men vs. women.

**Figure 3:**
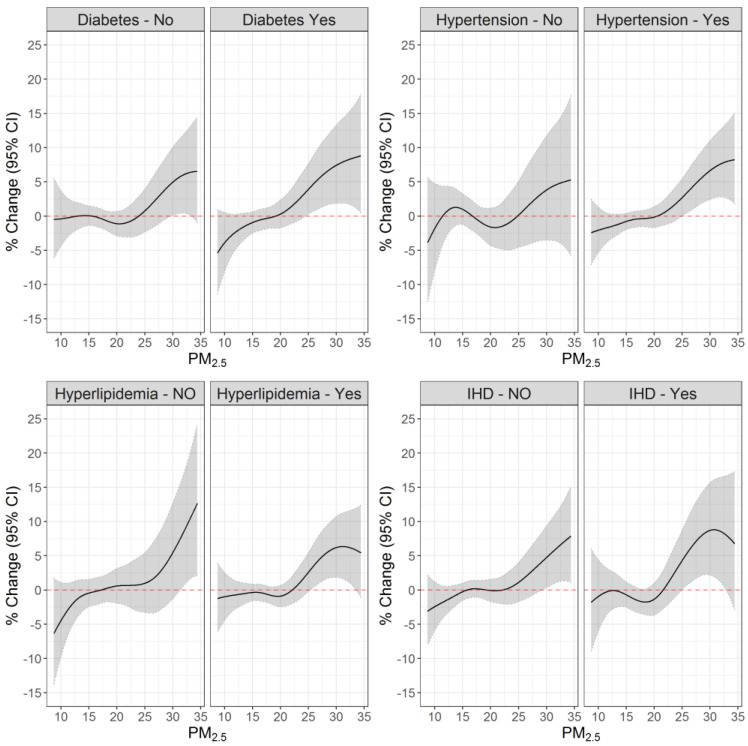
The association between PM_2.5_ exposure on the event day and ischemic stroke/TIA stratified by cardiometabolic chronic conditions. Figure 3 shows the associations between PM_2.5_ exposure on the event day and ischemic stroke/transient ischemic attack (TIA), stratified by diabetes, hypertension, hyperlipidemia, and ischemic heart disease (IHD).

The PM_2.5_ and ischemic stroke/TIA associations were also larger among individuals with diabetes, hypertension, and ischemic heart disease, compared to those without the disease (Figure 3). For example, setting PM_2.5_ exposure at a 30 µg/m^3^ reference, we found 7.43% (1.86%; 13.30%) and 4.96% (0.09%; 10.06%) increased risks among people with and without diabetes, 6.65% (2.44%; 11.03%) and 3.81% (−3.52%; 11.70%) among people with and without hypertension, and 8.70% (2.17%; 15.66%) and 4.74% (0.35%; 9.33%) among people with and without IHD.

## 4. Discussion

In this retrospective cohort study, we leveraged a population-based registry and PM_2.5_ exposure models at a fine spatiotemporal resolution to assess the association between PM_2.5_ exposure and stroke. While restricting our analysis to only days where average PM_2.5_ concentrations fell within the Israeli air quality standard of 37.5 µg/m^3^, we found an increased risk of ischemic stroke associated with PM_2.5_ exposure. Furthermore, this association was modified by sociodemographic factors and comorbidities, with larger effects observed among women, non-Jewish individuals, older adults, and individuals with diabetes, hypertension, and ischemic heart disease.

Our findings corroborate the existing literature that found associations between long-term and short-term particulate matter exposure and ischemic stroke among other cardiovascular morbidities. For example, a prospective cohort study conducted in China found that long-term PM_2.5_ exposure was associated with a hazard ratio of 1.53 among those exposed to levels higher than 78.2 µg/m^3^ compared to those exposed to lower levels [24]. A meta-analysis conducted by Yuan et al. observed a pooled hazard ratio of 1.11 for stroke at a 5 µg/m^3^ increase in PM_2.5_ in studies conducted in North America and Asia [10].

More importantly, we added evidence of harmful air pollution effects of exposure levels lower than national standards [25,26,27,28]. These standards are set to protect humans and the environment from any harmful consequences of air pollution exposure. Yet, adverse air pollution effects associated with air pollution levels within national standard were observed in many studies around the world. In the United States, while analyzing PM_2.5_ and ozone exposure within the National Ambient Air Quality Standards (NAAQS) set by the U.S. EPA, Quin Di et al. found that air pollution was still associated with higher risks of mortality among older adults [25]. A few other studies also conducted in the United States found similar results that indicated continued increased risks for cardiovascular disease morbidity and mortality at exposure levels lower than the NAAQS [27,29,30]. Wolf et al. conducted an analysis of the Effects of Low-Level Air Pollution: A Study in Europe (ELAPSE) cohort studies, which also found an increased risk for ischemic stroke associated with PM_2.5_ exposure even within the air quality standards [31]. In 2021, the World Health Organization (WHO) concluded that harmful air pollution effects on health were observed at even lower concentrations than previously thought, and markedly reduced their recommended air pollution levels [32]. The current guidelines suggest reducing 24 h mean PM_2.5_ levels below 15 µg/m^3^. The WHO guidelines are reflected in the Israeli Clean Air Act, and the interim target for daily PM_2.5_ levels is 15 µg/m^3^. The standard, however, is much higher (37.5 µg/m^3^). Our findings imply that current policies and standards attempting to safeguard populations from the harmful effects of air pollution exposure may not be enough to protect those at risk.

Regarding ICH, we initially observed a protective effect while using a linear term. However, as supported by the nonlinear exposure–response curve, there was no association with ICH and the protective effect could be attributed to larger errors in this smaller groups. The lack of association with ICH can be due to the lower occurrence of this type of stroke, or due to a different pathology. Similar to our findings, a recent meta-analysis concluded that the majority of studies evaluated did not find a significant association between ICH and short-term air pollution exposure [3]. A nonsignificant negative relationship between PM_2.5_ and ICH was observed in some studies which they attributed to the differences in the pathology of ICH and ischemic stroke [5,8,9].

An important finding of our study is the larger effects observed among women, older adults, non-Jewish individuals, and people with cardiometabolic chronic conditions. As with our findings, a few other studies found larger estimates among women [33,34]. However, more commonly, studies have reported risks that were either higher among males or similar across sex [3,35]. This could be due to a physiological difference in lung size among women that can lead to higher amounts of damage caused by particles [36].

As in our study, other studies have found larger susceptibility to air pollution health effects among the non-Jewish population [12,13]. A recent study which used the INSR to assess differences in stroke risk between Arab and Jewish patients found differences in baseline risks, with higher rates of smoking and diabetes among Arab patients. Treatment and survival outcomes, however, were similar between groups [37]. Additionally, other studies conducted in Southern Israel found higher air-pollution-related asthma rates among Bedouin Arab localities, where a large percentage of the population reside in temporary housing, suggesting a lower socioeconomic status and higher indoor air pollution infiltration [12,13]. Poor housing conditions and exposures to indoor environmental pollutants induce risk for adverse health outcomes [38]. The higher susceptibility observed in our study can possibly be attributed to higher exposure levels due to housing conditions, a higher prevalence of lower socio-economic status, or a higher prevalence of risk factors for stroke.

Similar to our findings, other studies have found larger air pollution effects on stroke among older individuals [3,39,40]. However, unlike our observations, a previous study conducted in the Southern part of Israel found a significant risk for ischemic stroke only among adult participants younger than 55 [41]. These differences may be related to the particle’s composition. The Southern part of Israel is a semi-arid region, with natural dust being a major source of air pollution [42]. Our study covered the entire country, and therefore included areas where traffic, industry, and other sources also contributed greatly to PM_2.5_ pollution. It may also be related to population characteristics that might be different in Southern Israel compared to other regions. Finally, as often seen in studies evaluating air pollution health consequences [14,17,43], we found people with cardiometabolic diseases to be more susceptible to the air pollution effect on the risk of stroke.

The major strength of this study is the use of a national cohort with comprehensive health information which allowed us to gain a better understanding of the association at a population level, including a broad population representing both rural and urban locations in an area with a unique climate and high concentrations of PM_2.5_ exposure. Our study also has several limitations. First, like other air pollution studies, we might have had an exposure misclassification error. However, due to the use of our highly spatiotemporally resolved exposure mode, this error is expected to be minimal [21]. Additionally, there may be potential residual confounding of unmeasured pollutants.

## 5. Conclusions

We found a higher risk of ischemic stroke associated with PM_2.5_ exposure with certain populations more vulnerable to PM_2.5_ exposure effects. This emphasizes the importance of not only behavioral health interventions in cardiovascular risk prevention, but population-level interventions to reduce exposure to air pollution. Even within the Israeli air quality standards, a higher risk of ischemic stroke is still present as exposure to PM_2.5_ increases. These findings support the importance of mitigating exposure to PM_2.5_ through policy and regulation. Policy and air quality standards must shift to integrate the growing evidence from research findings that bolster the associations between air pollution exposure and cardiovascular health in their continued decision-making.

## Figures and Tables

**Table 1:** Population characteristics (N = 74,052).

Sex	
Female	34,347 (46.4)
Male	39,705 (53.6)
Age	71.7 (13.6)
Ethnicity	
Jewish	59,900 (80.9)
Arab	11,217 (15.1)
Other	2940 (4.0)
Primary diagnosis	
Intracranial hemorrhage	5685 (7.7)
Ischemic stroke	47,959 (64.6)
Transient ischemic attack	20,413 (27.7)
Diabetes	32,543 (43.9)
Hypertension	56,896 (76.8)
Hyperlipidemia	52,341 (70.7)
Atrial fibrillation	15,258 (20.6)
Congestive heart failure	10,623 (14.3)
Ischemic heart disease	23,335 (31.5)
Obesity	7162 (9.6)
History of myocardial infarction	2164 (2.9)

**Table 2:** The association between an IQR increase in PM_2.5_ exposure and subtypes of stroke.

Outcome	% Change (95% CI)	*p* Value
Ischemic stroke		
Lag 0	1.70% (0.15%; 3.28%)	0.030
Lag 1	0.11% (−0.72%; 0.95%)	0.795
Lag 2	−0.36% (−0.91%; 0.18%)	0.196
ICH		
Lag 0	−4.43% (−8.57%; −0.09%)	0.045
Lag 1	−0.37% (−2.57%; 1.87%)	0.740
Lag 2	−0.26% (−1.66%; 1.16%)	0.717
TIA		
Lag 0	2.74% (0.37%; 5.17%)	0.023
Lag 1	−0.44% (−1.78%; 0.91%)	0.521
Lag 2	−0.52% (−1.37%; 0.33%)	0.231

Table 2 shows the associations between an interquartile range (IQR) increase in PM_2.5_ exposure (6.3 µg/m^3)^ at lag days 0–2 and intracerebral hemorrhage (ICH), ischemic stroke, or transient ischemic attack (TIA).

**Table 3:** A sensitivity analysis restricting the data to cases with exact addresses.

Outcome	Main Analysis	Restricted to Cases with Exact Addresses
Ischemic stroke	1.70% (0.15%; 3.28%)	1.42% (−0.36%; 3.25%)
ICH	−4.43% (−8.57%; −0.09%)	−4.52% (−9.33%; 0.52%)
TIA	2.74% (0.37%; 5.17%)	2.60% (−0.12%; 5.40%)

Table 3 shows the associations between an interquartile range (IQR) increase in PM_2.5_ exposure (6.3 µg/m^3)^ at lag day 0 and intracerebral hemorrhage (ICH), ischemic stroke, or transient ischemic attack (TIA) in the main dataset and a restricted data including the 71% of individuals with exact address information.

## Data Availability

Restricted by our Data Use Agreement with the Israeli Ministry of Health, the INSR data that support the findings of this study are neither sharable nor publicly available. Academic and nonprofit researchers who are interested in using the data should contact the Israeli Ministry of Health directly. The code used to analyze the data can be shared upon request.
